# Neurodegenerative fluid biomarkers are enriched in human cervical lymph nodes

**DOI:** 10.1093/brain/awae329

**Published:** 2024-10-21

**Authors:** Adam Al-Diwani, Nicholas M Provine, Andrew Murchison, Rhiannon Laban, Owen J Swann, Ivan Koychev, Fintan Sheerin, Sandro Da Mesquita, Amanda Heslegrave, Henrik Zetterberg, Paul Klenerman, Sarosh R Irani

**Affiliations:** Department of Psychiatry, University of Oxford, Oxford OX3 7JX, UK; Pandemic Sciences Institute, Nuffield Department of Medicine, University of Oxford, Oxford OX3 7DQ, UK; Department of Radiology, John Radcliffe Hospital, Oxford University Hospitals NHS Foundation Trust, Oxford OX3 9DU, UK; Fluid Biomarker Laboratory, UK Dementia Research Institute at UCL, London W1T 7NF, UK; Fluid Biomarker Laboratory, UK Dementia Research Institute at UCL, London W1T 7NF, UK; Department of Psychiatry, University of Oxford, Oxford OX3 7JX, UK; Department of Radiology, John Radcliffe Hospital, Oxford University Hospitals NHS Foundation Trust, Oxford OX3 9DU, UK; Department of Neuroscience, Mayo Clinic, Jacksonville, FL 32224, USA; Fluid Biomarker Laboratory, UK Dementia Research Institute at UCL, London W1T 7NF, UK; Department of Neurodegenerative Disease, UCL Institute of Neurology, Queen Square, London WC1N 3BG, UK; Fluid Biomarker Laboratory, UK Dementia Research Institute at UCL, London W1T 7NF, UK; Department of Neurodegenerative Disease, UCL Institute of Neurology, Queen Square, London WC1N 3BG, UK; Department of Psychiatry and Neurochemistry, Institute of Neuroscience and Physiology, The Sahlgrenska Academy at the University of Gothenburg, Mölndal 431 39, Sweden; Clinical Neurochemistry Laboratory, Sahlgrenska University Hospital, Mölndal 431 39, Sweden; Hong Kong Center for Neurodegenerative Diseases, Clear Water Bay, Hong Kong; Wisconsin Alzheimer’s Disease Research Center, University of Wisconsin School of Medicine and Public Health, University of Wisconsin-Madison, Madison, WI 53792, USA; Pandemic Sciences Institute, Nuffield Department of Medicine, University of Oxford, Oxford OX3 7DQ, UK; Translational Gastroenterology Unit, Nuffield Department of Medicine, University of Oxford, Oxford OX3 9DU, UK; Peter Medawar Building for Pathogen Research, Nuffield Department of Medicine, University of Oxford, Oxford OX1 3SY, UK; Department of Neuroscience, Mayo Clinic, Jacksonville, FL 32224, USA; Oxford Autoimmune Neurology Group, Nuffield Department of Clinical Neurosciences, University of Oxford, Oxford OX3 9DU, UK; Department of Neurology, Mayo Clinic, Jacksonville, FL 32224, USA

**Keywords:** dementia, biomarker, meningeal lymphatics, cervical lymph nodes

## Abstract

In animal models, brain neurodegeneration biomarkers drain into cervical lymph nodes (CLNs), and this drainage function is reduced with ageing. If this occurred in humans, CLNs may provide a readily accessible measure of this aspect of protein clearance. We tested this hypothesis in people using ultrasound-guided fine needle aspiration.

We measured amyloid-beta 40 and 42, phosphorylated tau 181 (pTau181), glial fibrillary acidic protein and neurofilament light using single molecule array in CLN aspirates and plasma from: (i) a discovery cohort of 25 autoimmune patients; and (ii) plasma, CLNs and capillary blood in four healthy volunteers, an optimization cohort.

Ultrasound-guided fine needle aspiration was well-tolerated by all participants. In both cohorts, all biomarkers were detected in all plasma and CLN samples, other than neurofilament light (8/17 of discovery cohort). CLN biomarker concentrations were significantly greater than plasma concentrations for all except neurofilament light, most markedly for pTau181 (266-fold; *P* < 0.02), whose CLN concentrations decreased with age (Spearman *r* = −0.66, *P* = 0.001).

This study presents the first evidence that neurodegenerative biomarkers are detectable in human CLNs. Raised CLN:plasma biomarker ratios suggest their concentration in CLNs may offer a distinct compartment for minimally-invasive measurement of brain clearance and lymphatic drainage, with potential applicability to study of ageing and future clinical trials.

## Teaser

Al-Diwani *et al.* show that biomarkers used in dementia diagnosis are present at higher levels in human cervical lymph node aspirates than in blood. This adds to evidence suggesting that cervical lymph nodes may be the first to receive drainage from the brain, and could allow minimally invasive assessment of brain clearance.

## Introduction

Neurodegenerative conditions are characterized by the accumulation of abnormal proteins in the brain. The pathological hallmarks of Alzheimer’s disease (AD) are amyloid-beta (Aβ) plaques and hyper-phosphorylated tau tangles.^1^ These, and other related neurodegenerative biomarkers, can be measured in CSF and blood, offering valuable insights into pathophysiology as well as clinical diagnosis, prognosis, and monitoring of AD and other dementias.

Understanding how these proteins move between different brain compartments and into peripheral systems may prove key to assessing their impaired clearance from the brain in dementias. Efforts largely focused on animal models have recently discovered glymphatic and meningeal lymphatic vascular systems as candidates for extracellular fluid and macromolecule efflux from the brain towards the periphery.^2-5^ This includes glymphatic efflux to the perivenous space as well as non-glymphatic CSF efflux from the subarachnoid space, both leading to meningeal lymphatics. Tracking CSF-injected molecules in mice, and intrathecal gadolinium-based contrast studies in humans, suggest this brain meningeal lymphatic outflow drains directly into the cervical lymph nodes (CLNs).^4,6-12^ Indeed, disruption of both upstream glymphatic efflux, such as through astrocytic AQP4 mislocalization, or downstream, at the level of meningeal lymphatic drainage into the CLNs in mice can accelerate brain Aβ plaque formation and even alter its clearance with anti-Aβ immunotherapy.^4,5,11,13^ Despite this clear rationale, it remains a major challenge to directly assay the biochemistry of this system in humans, due to its largely intracranial anatomy. However, CLNs are extracranial and, furthermore, likely convergent hubs in this drainage system. Therefore, we predicted that sampling of human CLNs would yield biomaterials to address this system.

Previously, we have accessed CLNs in human participants using ultrasound-guided fine needle aspiration (FNA). This has proven both safe and acceptable to our participants. The FNAs have successfully identified both CNS antigen-directed lymphocytes and lymph-node specific proteins with prominent roles in CNS biology^14-16^; both show the enrichment of brain-specific markers within CLNs. Here, we significantly extend these concepts to test the hypothesis that fluid biomarkers relevant to neurodegenerative processes are quantifiable in the CLNs, enriched compared to blood, and can be studied as proxies of meningeal drainage into CLNs. If so, sampling from this anatomical site has the potential to illuminate both brain physiology and to complement CSF and blood analyses for biochemical indices of clearance, particularly in experimental medicine and trial settings.

## Materials and methods

### Participants

A discovery cohort consisted of previously acquired frozen CLN aspirates and matched plasma samples from 25 participants with autoimmune neurological diseases (16 CLN aspirates and 23 plasma samples; median age 60 years, range = 22–84, 14 female; Supplementary Table 1).^14,15^ To study fresh samples and exclude potential disease and sampling method-related confounding, we prospectively recruited four healthy adult volunteers with no self-reported or formal diagnoses of cognitive impairment (optimization cohort: mean age 33 years, range 24–38; three male; Supplementary Table 2). All provided informed written consent to donate CLN aspirates and paired peripheral blood (venous and capillary) in accordance with the Declaration of Helsinki and ethical approval (Research Ethics Committee 16/YH/0013).

### Sampling

#### Cervical lymph node fine needle aspiration

All FNAs were performed by a senior radiologist in a clinical ultrasound suite, as previously described,^14-16^ between 8 and 9 a.m., without mandating fasting. In brief, CLNs were visualized under ultrasound guidance, and after skin sterilization, multiple passes were performed on the same node using a 23G hypodermic needle. Approximately 30 μl of material was obtained, and immediately after each needle withdrawal, ice-cold sterile PBS solution was aspirated through the needle bevel into a syringe, and the material was then ejected into a 1.5 ml microtube. The discovery cohort focused on cell recovery rather than supernatant optimization such that needle passes were repeated two to three times with varying volumes (0.5–2 ml) for each needle wash. These were pooled, resulting in variable total supernatant volumes (1.5–8 ml). In the optimization cohort, we standardized supernatant acquisition with exactly two needle passes, and the material was carefully washed with 1 ml PBS/wash each into a separate 1.5 ml microtube for each of the four washes per needle. In both cohorts, following transfer to the laboratory on ice, samples were centrifuged for 5 min (200*g*–400*g*), and supernatants were stored at −80°C.

#### Capillary blood

Capillary blood was also collected by lancet to sample a micro-vascularized tissue akin to the lymph node hilum, which lacked a specialized lymphatic confluence. One drop (∼30 μl) was collected from the lateral aspect of the pulp of the fourth digit into a 1.5 ml microtube and, as per the lymph node FNA procedure, aspirated into a 23G hypodermic needle followed by a 1 ml of ice-cold PBS pull-through. Thereafter, the needle was removed and the mixture ejected into a fresh 1.5 ml microtube. The diluted capillary blood was centrifuged at 400*g* for 5 min. The acellular supernatant was transferred to a fresh 1.5 ml microtube and stored at −80°C. The tube lacked anti-coagulant, but there was no visible clotting. We refer to the diluted capillary plasma as capillary blood supernatant.

#### Venous blood

Standard venepuncture was also conducted to collect venous blood in an EDTA tube for plasma, as used here, and for peripheral blood mononuclear cell isolation reported previously.^16^ Following transfer to the laboratory on ice, blood was layered over 15 ml lymphoprep and centrifuged at 931*g* for 30 min with no brake. Plasma was transferred to 1.5 ml microtubes and stored at −80°C.

#### Assays

The samples were assayed on the ultrasensitive Quanterix HD-X single molecule array (SIMOA) platform: a Human Neurology 4-Plex E (N4PE) kit for two isoforms of Aβ_40_ and Aβ_42_, glial fibrillary acidic protein (GFAP) and neurofilament light (NfL), and a monoplex phosphorylated tau-181 Advantage V2.1 kit (pTau181). To optimize assays of CLN and capillary supernatants, serial dilutions in sample buffer found a 2-fold dilution to be optimal. Raw concentrations were multiplied by this assay dilution factor. Each sample was run in duplicate and the mean reported. Concentrations were further multiplied by a correction factor to account for dilution at the time of sampling (dilution volume/material volume, which for the optimization cohort was 1000 μl/30 μl = 33.33).

### Statistical analysis

Analyses were performed in Excel v16.83 (Microsoft) and Prism v10.2.1 (GraphPad). Data were rounded to three significant figures to reduce pseudo-exactness given known immunoassay coefficient of variation (5%–10%). Paired data were compared using a Wilcoxon signed-rank test or a two-tailed paired Student’s *t*-test for non-normal and normally distributed data, respectively. Unpaired non-normally distributed data were compared using a two-tailed Mann–Whitney test. Correlation was estimated with Spearman’s rank coefficient.

## Results

**Figure 1:**
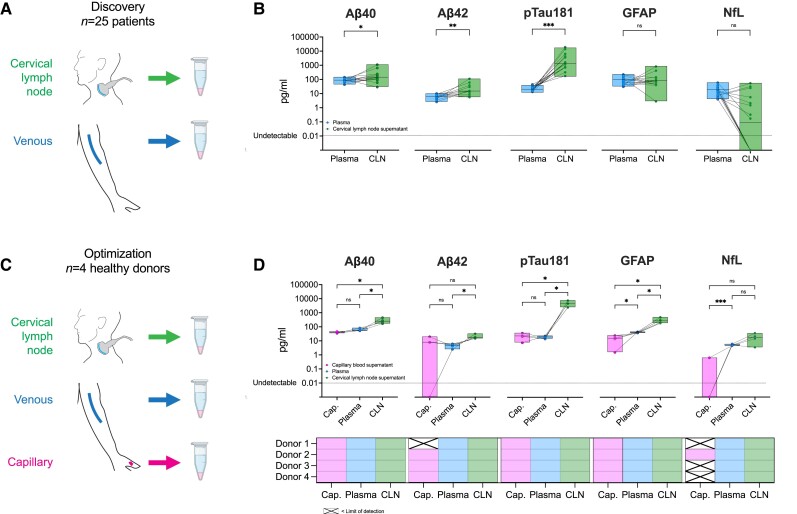
**Corrected concentrations of dementia fluid biomarkers in plasma versus capillary and cervical lymph node supernatants**. (**A**) Schematic diagram illustrates sample sources (venous blood as plasma and cervical lymph node aspirate supernatant) from 25 clinical donors with autoimmune neurological diseases, which acted as a discovery cohort. (**B**) Dot and box plots indicate the concentrations of five dementia fluid biomarkers from two colour-coded bio-samples from 25 individuals on a logarithmic axis (plasma = blue; cervical lymph node supernatant = green). The box spans the minimum to maximum value with a horizontal line at the median. Lines between dots indicate the same individual across the two sample types. A dotted line runs across the graphs to indicate the limit of assay detection. The result of a Wilcoxon signed-rank test for each protein is indicated above the plots. (**C**) Schematic diagram illustrates sample sources (venous blood as plasma, capillary bed blood as supernatant, and cervical lymph node aspirate supernatant) from four healthy donors, which acted as an optimization cohort. (**D**) Dot and box plots indicate the concentrations of five dementia fluid biomarkers from three colour-coded bio-samples from four individuals on a logarithmic axis (capillary blood supernatant = pink; plasma = blue, cervical lymph node supernatant = green). The box spans the minimum to maximum values with a horizontal line at the median. Lines between dots indicate the same individual across each sample type. A dotted line runs across the graphs to indicate the limit of assay detection. The result of a two-tailed paired *t*-test for each protein is indicated above the plots. The results for the above protein are summarized below for each donor and sample type. Cells are filled with the same colour scheme to indicate a positive result and ‘X' indicates that this was below the limit of detection. Aβ = amyloid-beta peptide; Cap. = capillary blood supernatant; CLN = cervical lymph node supernatant; GFAP = glial fibrillary acidic protein; NfL = neurofilament light; ns = not significant; pTau181 = phosphorylated tau protein 181. *P ≤ 0.05, **P ≤ 0.01, ***P ≤ 0.001. Illustrations in **A** and **C** are modified from Provine *et al*.^16^

All participants underwent sampling without any physical or psychological adverse events. In the discovery cohort, all five biomarkers were present in all of the CLN samples, with the exception of NfL which was detectable in 8/17 (Fig. 1A and B). The corrected CLN concentrations of Aβ_40_, Aβ_42_ and pTau181 were significantly higher than those in plasma (*P* = 0.03, 0.005 and <0.001, respectively, two-tailed Wilcoxon signed-rank test). For pTau181, the magnitude of difference was greatest: ∼65-fold, with a median CLN corrected concentration of 1465 pg/ml (range 176–18 900) versus a median plasma concentration of 22.6 pg/ml (range 13.3–43.6). No significant differences were noted for GFAP and NfL.

To reduce the impact of both pooling >1 needle pass and sample dilution, in the subsequent optimization cohort, we: (i) stored each wash from needle passes one and two separately; and (ii) reduced all samples to a constant resuspension volume of 1 ml. As expected, the diluted CLN samples showed greater variance than plasma. Furthermore, within CLN samples, the second FNA needle pass yielded more tightly distributed concentrations (Supplementary Fig. 1A and Supplementary Table 3). Specifically, the first wash from this second needle consistently showed the highest concentration compared to the first needle washes. Both observations are consistent with our prior proteomic data (Supplementary Fig. 1B and Supplementary Table 3).^16^ Using the first pass from the second needle, all biomarkers were detectable in all CLN supernatants and paired plasma in all four participants (Fig. 1C and D). In contrast, Aβ_42_ and NfL were undetectable in one and three of the four capillary supernatants, respectively. All CLN samples showed higher corrected concentrations of Aβ_40_, Aβ_42_, pTau181 and GFAP compared to plasma (*P* = 0.04, 0.05, 0.02 and 0.03, respectively, two-tailed paired *t*-test; Fig. 1D) and of Aβ_40_, pTau181 and GFAP compared to capillary supernatants (*P* = 0.03, 0.02 and 0.03, respectively, two-tailed paired *t*-test; Fig. 1D). There were no statistically significant differences between plasma and capillary blood for Aβ_40_, Aβ_42_ and pTau181, although GFAP and NfL were significantly lower in the capillary samples (*P* = 0.02 and 0.001, respectively, two-tailed paired *t*-test; Fig. 1D).

Consistent with the discovery cohort, the highest fold differences in corrected concentrations were between CLN compared to both plasma and capillary samples for pTau181: 266 times higher in CLN than plasma (mean 4865 versus 18.3 pg/ml; *P* = 0.02, two-tailed paired *t*-test), without differences between capillary and blood plasma (18.4 versus 21.8 pg/ml, ratio = 0.84; *P* = 0.65, two-tailed paired *t*-test; Fig. 1D). While the absolute mean corrected concentration of CLN Aβ_40_ was higher than Aβ_42,_ the ratio of each versus their respective plasma samples was similar (4.5) and the Aβ_42/40_ ratio, a commonly used dementia biomarker index, was not different between plasma and CLN (Supplementary Fig. 2).

**Figure 2:**
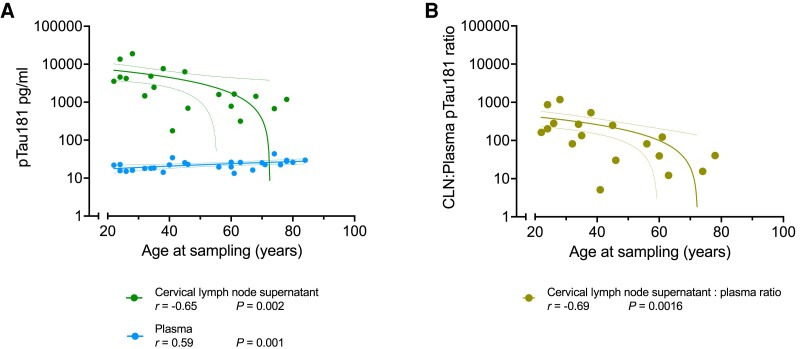
**Association of phosphorylated tau 181 (pTau181) concentration in cervical lymph node and plasma with age.** (**A**) Correlation of corrected concentration of pTau181 in cervical lymph node supernatant and plasma is plotted against age (cervical lymph node = green; plasma = blue). (**B**) Correlation of the ratio between corrected concentration of pTau181 in cervical lymph node supernatant to corresponding plasma ratio is plotted against age. Each dot is one sample or ratio, respectively; a line of best fit is drawn to summarize the relationship with dotted lines indicating the 95% confidence interval. The Spearman correlation coefficient (*r*) and statistical significance (*P*) for each line are given below the graphs. CLN = cervical lymph node.

In animal models, there is less lymphatic drainage of AD pathology-related proteins into CLNs with ageing. To explore this hypothesis in humans, we combined both groups to yield a broad age range (*n* = 29; median 56 years, range 22–84) and observed that plasma pTau181 positively correlated with age (Spearman *r* = 0.59, *P* = 0.001) but CLN levels showed a negative correlation of pTau181 versus age (Spearman *r* = −0.65, *P* = 0.002) (Fig. 2A). This relationship was also preserved when excluding the four samples from the optimization cohort (Spearman *r* = −0.62, *P* = 0.012) and when expressed as a ratio between CLN and plasma pTau181 levels (Spearman *r* = −0.69, *P* = 0.0016; Fig. 2B). The variation in CLN pTau181 concentration was not explained by variation in sample dilution (Spearman *r* = −0.11, *P* = 0.64; Supplementary Fig. 4). We did not detect a correlation between CLN and age in these data for the other four biomarkers, but the plasma levels of GFAP and NfL positively correlated with age (Spearman *r* = 0.69, *P* < 0.0001 and *r* = 0.81, *P* < 0.0001, respectively; Supplementary Fig. 3).

## Discussion

Our results show that CLN aspirates harbour concentrated levels of proteins that are currently used as neurodegenerative biomarkers. Given the direct and intimate connections between the brain and CLNs, mediated by meningeal lymphatics, we propose CLNs are likely to represent a source of brain drainage-related material. These findings are consistent with the marked enrichment of other CNS proteins we observed in CLN aspirations.^16^ The ultrasound-guided FNA methodology was performed by expert radiologists and proved both safe and minimally invasive, comparing favourably in these terms to the widely-used practice of lumbar puncture for CSF sampling. The information obtained from these two sample types is of course fundamentally different, and thus we propose CLN sampling could be a valuable complementary research tool to investigate the clearance of proteins from the brain to the periphery in health and disease, including in studies of neurodegeneration.

In the technically standardized younger adult optimization group, CLN biomarker levels also showed higher concentrations than blood. This suggests these proteins are being physiologically concentrated in CLNs, rather than reflecting blood contamination. This anatomical compartmentalization is consistent with clear distinctions in cellular populations we have previously observed when comparing paired samples of CLNs and blood, such as enrichment for lymph node resident T follicular helper cells and relative exclusion of monocytes.^14-16^

The next critical question is whether these proteins are being enriched in CLNs via brain lymphatic drainage, non-brain head and neck drainage, local production, or a combination. One clue comes from the relative neural specificity of some of the proteins described here. In our prior study, gene ontology commonly ascribed neural functions to CLN-restricted proteins. Examples included secernin-1, a tau-binding protein,^17^ the key microglial receptor TREM2, and ADAM22, a key receptor of the neuronal autoantibody target LGI1.^18^ It may also be that local lymph node sources are relevant. For example, TREM2 is expressed by lymph node sub-capsular macrophages,^19^ and both Aβ and phosphorylated tau can be found in the periphery, including in the head and neck.^20,21^ Nonetheless, as exemplified by a study of post-stroke tonsillar biopsies, these regions themselves can be differentially enriched for drained brain-derived proteins.^22^ Moreover, as several independent animal models have shown, both dynamically and at post-mortem, the CLNs directly receive CSF brain drainage from the meningeal lymphatics.^7-9^ Indeed, a recent study comparing the proteome of cannulated lymph in cervical versus mesenteric chains showed distinct patterns with brain protein networks highly represented in CLNs.^23^ Future studies could include both other lymph node basins and concomitant CSF sampling. In combination with these complementary prior studies, we predict that biomarker concentrations detected in the CLNs may better reflect brain CSF levels than plasma, given the high and constant rate of CSF macromolecule drainage into the CLNs via meningeal lymphatics.^3-5,12^

The varied profiles of these biomarkers in CLNs merit consideration of their biological functions in the brain and periphery. Most striking was the marked concentration difference of pTau181 in CLN versus blood. pTau181 in blood and CSF is associated with the clinical phase of Alzheimer’s disease and cerebral atrophy,^24^ yet the optimization group donors were young with no self-reported or formal diagnoses of cognitive impairment. While tau is an intracellular protein, secreted abnormal forms may propagate through synaptic networks in AD in a prion-like process, and via secreted extracellular vesicles, hence rendering extracellular tau clearance pathophysiologically important.^7,25-28^ Our data could be compatible with a model including constitutive drainage of phosphorylated tau species into the CLNs via glymphatic and lymphatic mechanisms that reduces with age, leading to greater tau accumulation in the extracellular compartment. Speculatively, this could then contribute to the secondary tauopathy manifesting as AD progression. However, further analysis of paired CLN-CSF samples examining the extensive range of tau isoforms, beyond one phosphorylated species, will be required to interrogate this notion further.

Yet, despite their detection at higher concentrations in the CLNs, neither Aβ_40_ nor Aβ_42_ showed such a similar magnitude difference between the two sites, nor did the CLN concentrations correlate with age. The Aβ precursor protein is widely expressed across extra-cerebral tissues, including blood platelets, myocytes and hepatocytes, from which both Aβ_40_ and Aβ_42_ are proteolytically produced and released.^29^ Aβ peptides have a high propensity for extracellular aggregation in the form of plaques, which form at a faster rate both in the brain and meninges in ageing AD transgenic mice. The aggregation propensity of Aβ might preclude its presence in the CSF that reaches the dura for drainage by the meningeal lymphatic vessels. It may also be that Aβ peptide molecules are cleared by a cellular route. Indeed, a histological study of pathological lymph nodes showed Aβ-staining cells were considerably more common in the cervical than the inguinal lymph nodes.^30^ Moreover, it may be that only a certain proportion of specific brain-derived proteins drain from meningeal lymphatics into the CLNs and so certain proteins may show alternative patterns. In contrast, the lack of difference for NfL is more explicable since elevated extracellular NfL demarcates neuro-axonal damage usually found in aggressive neuro-degenerative or neuro-inflammatory processes. Thus, the lack of elevated NfL in the CLNs is consistent with the profile of the donors.

Limitations include the small numbers in our optimization cohort, the potential confound of varied autoimmune neurological conditions and differences in sample preparation. Indeed, the presence of donors with AQP4-antibodies raises whether glymphatic mechanisms were involved, which could be studied specifically in future. Nonetheless, the major data trends appear consistent across the two independent cohorts, analytic variation does not explain the age correlation observed in the discovery cohort, and older participants did not just include those with prior NMOSD. Moreover, as the age-related correlation is only seen with pTau181, sample artefact seems unlikely. Yet, the level of variance in sample observations suggests there is further scope for sample optimization, and our existing methods outline areas that could benefit from refinement. Alongside suggesting modest sample dilutions are adequate, we highlight the differential concentrations of these biomarker proteins between an initial and second needle pass—consistent with our observations at proteomic scale.^16^ We propose this reflects some tissue dissociation following the first needle pass in CLNs, which is then more easily aspirated in the second pass, also mirrored with sample white cell count.

The large effect sizes seen in this pilot study, together with our previous immunologic and proteomic data, suggest we are accessing a wealth of brain-relevant material in human CLNs, towards exploring pathobiology and biomarker discovery. Unlike in animal models, the precise cannulation of afferent or efferent lymphatic vessels in humans is unlikely to be feasible. However, when relating a bodily fluid (plasma, CSF) to a resuspended tissue puncture (CLN FNA), comparable quantifications will allow drainage-based hypotheses of protein clearance to be investigated. At this stage, while we report initial absolute values, the main observation is the relative fold differences between compartments. This system will be fully elucidated with sampling of other lymph node basins, CSF and imaging indices. To help define exact normative values, larger cohort sizes encompassing both healthy ageing and people with AD pathology and dementias will be required. This will be particularly interesting at extremes of the AD continuum, such as ‘super-agers’, including those who are cognitively resilient to AD pathology as well as those who are highly resistant to developing the pathology at all. Our approach will allow the contribution of drainage preservation to be tested.

In conclusion, we provide independently validated evidence to support the presence of dementia fluid biomarkers in CLNs, in all except NfL, at significantly higher levels than circulating plasma. Moreover, we provide the first evidence in humans of reduced brain lymphatic drainage with age of the disease-relevant biomarker pTau181. Taken together, these observations likely reflect potential to longitudinally quantify *in vivo* lymphatic drainage of brain disease biomarkers from CLN aspirates. Hence, this minimally invasive technique is likely of great value for evaluating brain protein clearance in human experimental medicine studies and early phase clinical trials. Given the relative ease and excellent safety profile of CLN FNA, we propose the technique is now ready to move from the rare autoimmune neurological diseases in which we pioneered its application to researching healthy ageing and more common brain disorders such as infection, traumatic brain injury and dementia.

## Supplementary Material

awae329_Supplementary_Data

## Data Availability

Data supporting the study are available from the corresponding authors upon reasonable request.
